# Consolidated Bioprocessing for Butyric Acid Production from Rice Straw with Undefined Mixed Culture

**DOI:** 10.3389/fmicb.2016.01648

**Published:** 2016-10-24

**Authors:** Binling Ai, Xue Chi, Jia Meng, Zhanwu Sheng, Lili Zheng, Xiaoyan Zheng, Jianzheng Li

**Affiliations:** ^1^Haikou Experimental Station, Chinese Academy of Tropical Agricultural SciencesHaikou, China; ^2^State Key Laboratory of Urban Water Resource and Environment, Harbin Institute of TechnologyHarbin, China

**Keywords:** butyric acid production, rice straw, consolidated bioprocessing, undefined mixed culture, carboxylate platform

## Abstract

Lignocellulosic biomass is a renewable source with great potential for biofuels and bioproducts. However, the cost of cellulolytic enzymes limits the utilization of the low-cost bioresource. This study aimed to develop a consolidated bioprocessing without the need of supplementary cellulase for butyric acid production from lignocellulosic biomass. A stirred-tank reactor with a working volume of 21 L was constructed and operated in batch and semi-continuous fermentation modes with a cellulolytic butyrate-producing microbial community. The semi-continuous fermentation with intermittent discharging of the culture broth and replenishment with fresh medium achieved the highest butyric acid productivity of 2.69 g/(L· d). In semi-continuous operation mode, the butyric acid and total carboxylic acid concentrations of 16.2 and 28.9 g/L, respectively, were achieved. Over the 21-day fermentation period, their cumulative yields reached 1189 and 2048 g, respectively, corresponding to 41 and 74% of the maximum theoretical yields based on the amount of NaOH pretreated rice straw fed in. This study demonstrated that an undefined mixed culture-based consolidated bioprocessing for butyric acid production can completely eliminate the cost of supplementary cellulolytic enzymes.

## Introduction

Butyric acid is a widely applied material in the chemical, textile, food and pharmaceutical industries (Zhang et al., 2009) with potential application in the production of biofuel butanol (Richter et al., 2012; Lee et al., 2014) and biodegradable plastics (Albuquerque et al., 2011). The industrial-scale production of butyric acid is currently accomplished by petrochemical processes (Dwidar et al., 2012). Butyric acid fermentation from renewable biomass may provide a reasonable alternative. However, because of the increasing price of fermentation feedstocks, namely corn and molasses, traditional butyric acid fermentation is not economically attractive. Using lignocellulosic biomass as the fermentation feedstock provides environmental and cost benefits, although the benefits gained from the low feedstock cost is completely counteracted by the cost of cellulolytic enzymes. In a sugar platform, the best-known biorefinery platform, cellulolytic enzymes are required to break down lignocelluloses into five- and six-carbon sugars that are further converted to desired chemicals, for example, ethanol (Agler et al., 2011). By contrast, purified cellulolytic enzymes are not necessary for lignocellulose conversion through a carboxylate platform, another biorefinery platform. Using a carboxylate platform, organic feedstocks are anaerobically converted by undefined mixed cultures into short-chain carboxylates (including acetate, propionate, lactate, and butyrate) as intermediate feedstock chemicals (Agler et al., 2011). By employing an undefined mixed culture as the source of fermentation organisms, the cost of added enzymes is completely eliminated, and it becomes possible to convert cellulosic feedstocks into the desired products in a single process termed consolidated bioprocessing (CBP). In CBP, cellulolytic enzyme production, both cellulose and hemicellulose hydrolysis, and fermentation to obtain desired products are combined in a single process step (Olson et al., 2012). CBP is the ultimate low-cost configuration for cellulose hydrolysis and fermentation because the highly integrated process requires less feedstock processing, lower energy inputs and yields a higher conversion efficiency (Fu and Holtzapple, 2010). The carboxylate platform-based MixAlco process, an example of CBP, employs a mixed culture of acid-forming microorganisms to convert lignocellulosic biomass to carboxylates which are subsequently chemically converted to other chemical and fuel products (Granda et al., 2009; Holtzapple and Granda, 2009). In the MixAlco process, sugarcane bagasse (Fu and Holtzapple, 2011), corn stover (Chan et al., 2011), office paper and pineapple residue (Forrest et al., 2010) with chicken manure as a co-substrate have been used for carboxylate production, primarily acetate. After inoculation with a mixed culture of marine microorganisms, a yield of 14.6–56.1 g/L of total carboxylic acids, of which 65.9 to 90.6% was acetic acid, was produced from a mixed feed of 80% lime-treated sugarcane bagasse with 20% chicken manure (Fu and Holtzapple, 2010).

Inspired by the carboxylate platform and CBP, undefined mixed culture-based fermentation technology is a promising alternative strategy for butyric acid production from lignocellulosic biomass. In a previous study, an anaerobic microbial community with stable cellulose-degrading potential and high selectivity for butyric acid production was selected from a combination of cattle manure, pig manure compost, corn field soil and rotten wood (Ai et al., 2013). To further demonstrate the feasibility and potential of undefined mixed culture for butyric acid production, a CBP process was investigated to produce butyric acid from NaOH pretreated rice straw using a cellulose-degrading butyric acid-producing microbial community, and its fermentation performance in batch and semi-continuous operations was evaluated.

## Materials and methods

### Experimental set-up

**Figure 1:**
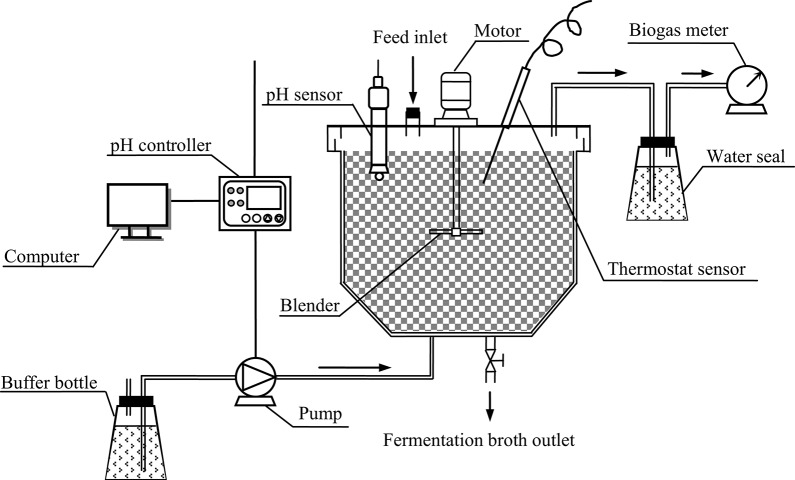
**Schematic diagram of the stirred-tank reactor system for butyric acid fermentation**.

A stirred-tank reactor with a working volume of 21 L was constructed for butyric acid fermentation (Figure 1). The fermentation temperature was maintained at 35 ± 1°C by wrapping heating wire around the reactor. A wet gas meter was used for the measurement of biogas volume. The water seal and wet gas meter were filled with acidified water (pH 3) to prevent the dissolution of CO_2_ contained in the biogas. The fermentation pH was controlled from 6.0 to 6.2 with saturated NaHCO_3_ solution as the buffer.

### Sodium hydroxide pretreatment of rice straw

Sodium hydroxide pretreatment is performed for efficient utilization of rice straw, the substrate for butyric acid fermentation. NaOH pretreatment is recognized as an effective method for delignification as well as swelling of biomass to increase digestibility. The was cut into 10- to 15-cm lengths and soaked in a 1% NaOH solution with a solid-to-liquid ratio of 1:15 (w/v) at 50°C for 72 h in static state. The solid residue was then separated by filtering and thoroughly washed with tap water to near-neutral pH. The neutralized residue was squeezed and stored at 4°C. The substrate contained 53.0% cellulose, 27.4% hemicellulose and 8.0% lignin. The untreated rice straw contained 39.7% cellulose, 24.8% hemicellulose and 15.3% lignin. The cellulose, hemicellulose and lignin were measured as previously described (Van Soest et al., 1991).

### Preparation of inoculum

The cellulolytic butyrate-producing mixed culture was derived from cattle manure, pig manure compost, corn field soil and rotten wood (Ai et al., 2013). The cattle manure, pig manure compost and corn field soil were collected from the suburb of Harbin, China. The rotten wood was collected from the campus of Northeast Forestry University, Harbin, China. All samples were taken in May, 2012 at locations 10 to 20 cm below the surface. The mixed culture was selected as a producer of butyric acid for its stable cellulose-degrading potential and high selectivity of butyric acid production from NaOH pretreated rice straw. The mixed culture includes cellulolytic and xylanolytic bacteria, butyrate-producing bacteria and other acidogenic bacteria. For the inoculum preparation, the stored mixed culture was transferred to seed medium which was composed of 10 g pretreated rice straw, 5 g tryptone, 1 g yeast extract, 5 g NaCl, 2 g CaCO_3_, and 0.5 g D-cysteine hydrochloride per liter, and one filter paper strip (1.5 × 5 cm) as an indicator. The broth was purged with nitrogen gas for 10 min to maintain anaerobic conditions, after which the 500-mL serum bottle containing 300 mL of broth was sealed and autoclaved at 115°C for 20 min. Following inoculation with 10 mL of the stored culture, the bottle was incubated at 35°C without agitation until the filter paper strip was broken down.

### Butyric acid fermentation procedure

The butyric acid fermentation medium was composed of 90 g pretreated rice straw, 5 g tryptone, 1 g yeast extract, 5 g NaCl, 6 g CaCO_3_, 0.5 g D-cysteine hydrochloride and 0.08 g chloroform per liter. Chloroform was used as an inhibitor of methanogenic bacteria. The fermentation reactor and medium were prepared without N_2_ purging and autoclaving. Fermentations were initiated by inoculating 1 L inoculum into 20 L of fermentation medium. In batch operation, aside from the NaHCO_3_ buffer, no additional medium ingredients were added to the fermentation system after the initial charge. For the semi-continuous operation, on the fourth day and every day thereafter throughout the fermentation period, 3 L fermentation broth was discharged and then 3 L fresh fermentation medium containing only 270 g pretreated rice straw and 15 g tryptone was added to the system.

### Analytical methods

The daily biogas production volume was monitored using a wet gas meter (LML-1, Changchun Automotive Filter Co., LTD., Changchun, China). Hydrogen, methane and carbon dioxide in the biogas were analyzed using a gas chromatography (SP-6800A, Lunan Instrument Factory, Shandong, China) equipped with a thermal conductivity detector and a 2 m stainless column packed with Porapak Q (60/80 mesh, ZhongKeKaiDi Chemical New-tech Co., Ltd., Lanzhou, China). Carboxylic acids in the fermentation broth were measured using a gas chromatography (SP-6800A, Lunan Instrument Factory) equipped with a flame ionization detector and a FFAP capillary column (30 m × 0.32 mm × 0.50 μm, ZhongKeKaiDi Chemical New-tech Co., Ltd.). Microbial biomass was estimated by optical density at 260 nm of fermentation broth after HClO_4_ hydrolysis as follows: To 5 mL of the culture broth, 5 mL of 1 mol/L HClO_4_ solution was added. If required, the samples were diluted to adjust the optical density within the readable range. The tubes were placed in boiling water for 20 min and cooled to room temperature. The contents of the tubes were centrifuged and the OD of the supernatant was measured at 260 nm. The OD_260_ of cultured broth was denoted by [A]. Uncultured broth was used as a blank, and the OD_260_ was denoted by [B]. The microbial biomass was denoted as [A]–[B]. The detailed operating conditions for determination of biogas composition, carboxylic acids and microbial biomass were as described previously (Ai et al., 2014b).

### Statistical analysis

Statistical analysis was performed using Statistical Product and Service Solutions (IBM SPSS Statistics for Windows, Version 19.0, IBM Corp., New York, US).

## Results and discussion

### Butyric acid production by batch fermentation

**Figure 2:**
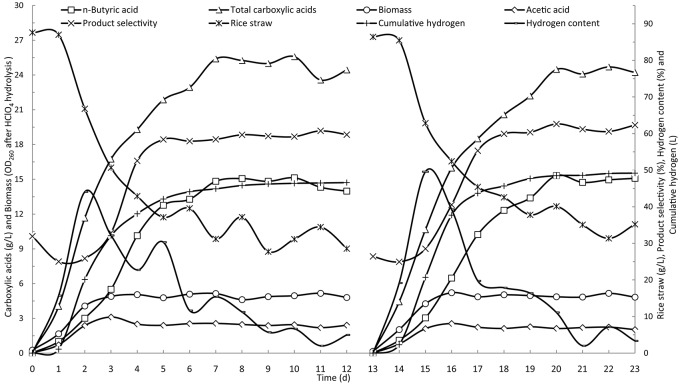
**Time courses of butyric acid production by batch fermentation in a stirred-tank reactor**.

Two batches of butyric acid fermentation in batch operation were conducted with run times of 10 and 12 days. Figure 2 shows the time courses of the batch fermentation of butyric acid from rice straw. The two batches of butyric acid fermentation followed a similar pattern, which verified the reproducibility of the mixed culture-based fermentation for butyric acid. Acetic and butyric acids were the main products in the fermentation broth from the eight kinds of carboxylic acids detected. The acids found at low concentrations are not shown in the figure. The fermentations underwent a 1-day lag phase followed by the rapid degradation of rice straw over the following 3 days. Similar to the degradation of rice straw, the carboxylic acids and hydrogen production increased from the second day and leveled off from the sixth day onwards. The acetic acid concentration peaked on the third day and then began to decrease the next day. The changes in butyric acid concentration lagged a day behind those observed for acetic acid, increasing rapidly on the third and fourth days. From the plot of product selectivity (percent of butyric acid from the total carboxylic acids) vs. time, it was evident that acetic acid was the primary carboxylic acid produced in the first 2 days with a product selectivity of about 25%. Over the following several days, the product selectivity increased to and was maintained at around 60%, indicating that butyric acid was the major product of the fermentation.

The cell biomass represented by the OD_260_ of the fermentation broth after HClO_4_ hydrolysis reached its maximum weight in the first 3 days, with little change over the remaining fermentation period. During the primary stage of fermentation, a large amount of hydrogen was generated in the reactor. The peak production of 20 L/d was observed on the second day and then quickly decreased. Hydrogen is a clear and sustainable energy source (Cheng et al., 2011). Additionally, as a reducing agent, hydrogen can be used to reduce carboxylic acids to alcohol fuels via chemical post-processing reactions, including ketonization and hydrogenation (Agler et al., 2011).

### Butyric acid production by semi-continuous fermentation

**Figure 3:**
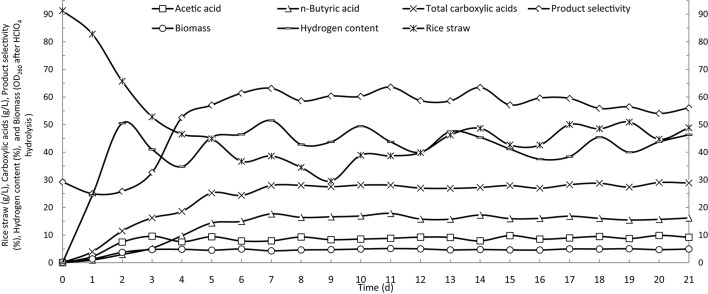
**Time courses of butyric acid production by semi-continuous fermentation in a stirred-tank reactor**.

**Figure 4:**
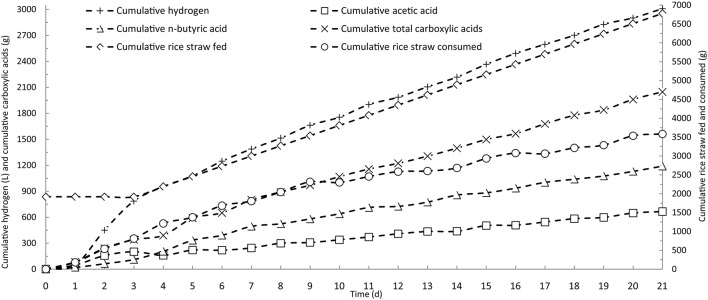
**Time courses of cumulative carboxylic acids and hydrogen by semi-continuous fermentation in a stirred-tank reactor**.

In batch operation, the consumption of rice straw and production of butyric acid reached their maximum rates on the fourth day of fermentation, and essentially decreased to zero after 7 days (Figure 2). Based on the results of batch fermentation, semi-continuous fermentation was implemented by discharging 3 L of fermentation broth and then feeding with 3 L of fresh fermentation medium from the fourth day and every day thereafter throughout the fermentation period. The detailed fermentation course of butyric acid production using the semi-continuous operation is presented in Figure 3. On the fourth day, the consumption of rice straw began to decrease. Upon the addition of fresh fermentation medium, the substrate consumption rate on the fifth day rebounded to 14.0 g/(L· d) from 7.4 g/(L· d) on the fourth day. However, from the tenth day onwards, the rice straw remaining in the fermentation broth continued to increase, indicating that substrate consumption decreased, likely as a result of strain degeneration and productivity decline. The total carboxylic acid production slowed down on the fourth day, but sharply increased on the fifth day when fresh substrates were fed to the fermentation system, whereafter, it remained stable with slight fluctuations. The butyric and acetic acid production followed similar changes in productivity over time. However, overall a decreasing trend was observed from the product selectivity curve. Over the last several days, the percent of butyric acid from the total carboxylic acids dropped below 60%. The aforementioned pattern of acetic acid formation, increasing on the second and third days and then decreasing to the basal level, was more clearly observed in the time course of cumulative acetic acid production presented in Figure 4. As shown from the curves of cumulative hydrogen production and hydrogen content, hydrogen generation was generally stable.

### Performance of batch and semi-continuous fermentation for butyric acid production

Table 1 summarizes the results of batch and semi-continuous fermentation for butyric acid. In the two batches of batch fermentation, 513.2 and 508.6 g of total carboxylic acids were produced, including 160.4 and 134.8 g of butyric acid. Because of the longer residence time of the substrate, the degradation rate of rice straw in the batch fermentation (63.4 and 58.9%) was higher than that in the semi-continuous fermentation (53.4%). The selectivity of butyric acid production and butyric acid yield in the batch fermentation were higher than those in the semi-continuous fermentation. The microbial cell biomass and carboxylic acid yield were not significantly different in the two fermentation operations. On the contrary, by employing a semi-continuous operation, the reactor volumetric productivity was significantly improved. For example, the butyric acid productivity was increased from 1.68 to 1.90 g/(L·d) in batch fermentation to 2.69 g/(L·d) in semi-continuous fermentation, and the carboxylic acid productivity from 2.83 to 3.08 g/(L·d) to 4.64 g/(L·d) (Table 1). The structure of rice straw and its solid state in the fermentation broth determine the efficiency of the biodigestion, and hence, inefficient biodigestion would yield a low utilization rate. As the fermentation progressed, the increasing lignin content made cellulose and hemicellulose more difficult to utilize, and the increasing carboxylic acids concentration aggravated product inhibition. Additionally, as a consequence of constantly feeding the NaHCO_3_ solution to buffer the pH, the sodium ion concentration in the fermentation broth continued to increase, which also inhibited the substrate utilization and carboxylic acid production. High Na^+^ concentration affects adenosine triphosphate content and activities of microbial enzymes, such as dehydrogenase and alkaline phosphatase (Hao et al., 2006). In the semi-continuous fermentation, by partly discharging the fermentation broth and then recharging with fresh fermentation medium, inhibition from end-products and Na^+^ can be minimized and the aging of carboxylic acid-fermenting strains can be extended. Furthermore, the semi-continuous fermentation operation shortened the nonproductive time and enhanced both efficiency and productivity. In this study, semi-continuous operation was observed to be more suitable for butyric acid production with an undefined mixed culture.

**Table 1:** **Summary of butyric acid production by batch and semi-continuous fermentation in a stirred-tank reactor**.

	**Batch fermentation 1**	**Batch fermentation 2**	**Semi-continuous fermentation**
Fermentation period (d)	12	10	21
Solids residence time (d)^a^	12	10	7
Cumulative acetic acid (g)	160.4	134.8	665.3
Cumulative propionic acid (g)	19.9	17.5	64.5
Cumulative *i*-butyric acid (g)	6.7	11.3	44.6
Cumulative *n*-butyric acid (g)	293.6	316.9	1189.4
Cumulative *i*-valeric acid (g)	13.2	12.7	39.9
Cumulative *n*-valeric acid (g)	6.5	4.3	14.7
Cumulative *i*-caproic acid (g)	11.0	9.7	26.8
Cumulative *n*-caproic acid (g)	1.8	1.3	2.5
Cumulative total carboxylic acids (g)	513.2	508.6	2047.7
Degradation rate of rice straw (%)	63.4 ± 5.4 A^c^	58.9 ± 5.1 A	53.4 ± 0.9 B
Product selectivity (%)^b^	59.4 ± 0.3 A	60.9 ± 0.3 B	58.1 ± 0.4 C
Biomass (OD_260_ after HClO_4_ hydrolysis)	5.01 ± 0.05 A	4.94 ± 0.2 A	4.92 ± 0.17 A
Butyric acid yield (g/g rice straw fed)	0.171 ± 0.002 AB	0.174 ± 0.003 A	0.167 ± 0.001 B
Carboxylic acids yield (g/g rice straw fed)	0.29 ± 0.00 A	0.28 ± 0.00 A	0.29 ± 0.00 A
Cumulative H_2_ yield (mL/g rice straw fed)	26.33 ± 0.17 A	27.8 ± 0.2 B	21.6 ± 0.3 C
Butyric acid productivity (g/L/d)	1.68 ± 0.19 A	1.90 ± 0.27 A	2.69 ± 0 B
Carboxylic acids productivity (g/L/d)	2.83 ± 0.3 A	3.08 ± 0.38 A	4.64 ± 0.03 B
H_2_ productivity (L/L/d)	0.26 ± 0.03 A	0.31 ± 0.04 AB	0.35 ± 0.01 B

a Solids residence time = Total working volume of fermentor/Flow rate of liquid out of fermentor.

b Percent of butyric acid from the total carboxylic acids.

c Values are expressed as mean ± SEM (n = 3). In batch fermentation, each value represents the mean of the determinations of the 7th, 8th, and 9th days, and in semi-continuous fermentation, the last three days. Values in the same row followed by the same capital letter are not significantly different at P = 0.05, according to Duncan's multiple range test.

### Theoretical potential of rice straw for butyric acid production

Cellulose is hydrolyzed to glucose, an intermediate in anaerobic digestion, and hemicellulose to xylose with small amounts of arabinose, mannose, and galactose. For the convenience of calculation, hemicellulose is modeled as xylan in this study. Equations (1, 2) show the conversion reactions of cellulose and hemicellulose polymers to monomers.

(1) $${\left({\text{C}}_{6}{\text{H}}_{10}{\text{O}}_{5}\right)}_{\text{n}}\;+\text{ n }{\text{H}}_{2}\text{O}\to \text{n }{\text{C}}_{6}{\text{H}}_{12}{\text{O}}_{6}$$

(2) $${\left({\text{C}}_{5}{\text{H}}_{8}{\text{O}}_{4}\right)}_{\text{n}}\;+\text{ n }{\text{H}}_{2}\text{O}\to \text{n }{\text{C}}_{5}{\text{H}}_{10}{\text{O}}_{5}$$

In anaerobic environments, during the primary fermentation with an undefined mixed culture, the five- and six-carbon sugars are converted to acetic acid and ethanol (C2), propionic acid and lactic acid (C3) and butyric acid (C4), which can be converted further by secondary fermentation to medium-chain carboxylic acids, for example, caproic acid (C6) and caprylic acid (C8) (Agler et al., 2011). Glucose is metabolized to pyruvate via the Embden–Meyerhof–Parnas (EMP) pathway (Agler et al., 2011), and xylose via the pentose phosphate pathway (PPP) (Temudo et al., 2009). The simplified Equations (3, 4) present the metabolic reactions of glucose to acetic acid and butyric acid, respectively.

(3) $$\begin{array}{r} \text{Glucose}\to 2\text{ Pyruvate}\to {2\text{ Acetate }+\;4\text{ H}}_{2}{\;+\;2\text{ CO}}_{2}\;+\;4\text{ ATP} \end{array}$$

(4) $$\begin{array}{r} \text{Glucose}\to 2\text{ Pyruvate}\to {\text{Butyrate }+\;2\text{ H}}_{2}{\;+\;2\text{ CO}}_{2}\;+\;3\text{ ATP} \end{array}$$

The simplified Equations (5, 6) present the metabolic reactions of xylose to acetic acid and butyric acid, respectively.

(5) $$\begin{array}{rcl} 3\text{ Xylose}\to 5\text{ Pyruvate} & \to & {5\text{ Acetate }+\;10\text{ H}}_{2}\\ +\;{5\text{ CO}}_{2}\;+\;10\text{ ATP} \end{array}$$

(6) $$\begin{array}{rcl} 3\text{ Xylose}\to 5\text{ Pyruvate} & \to & {2.5\text{ Butyrate }+\;5\text{ H}}_{2}\\ +\;{5\text{ CO}}_{2}+7.5\text{ ATP} \end{array}$$

Based directly on Equations (1, 3), 0.74 g of acetic acid can be obtained from 1 g of cellulose; based on Equations (2, 5), 0.76 g of acetic acid can be obtained from 1 g of hemicellulose (defined as xylan). Based on Equations (1, 4), 0.54 g of butyric acid can be obtained from 1 g of cellulose; based on Equations (2, 6), 0.56 g of butyric acid can be obtained from 1 g of hemicellulose. According to the calculation results above, 0.60 g of acetic acid or 0.43 g of butyric acid, theoretically, can be produced from the NaOH pretreated rice straw used in this study as it contained 53.0% cellulose and 27.4% hemicellulose.

### Actual yield of butyric acid production from rice straw by undefined mixed culture

Based on the fermentation results from the batch and semi-continuous operations, the ratios of actual yield to theoretical yield of butyric acid production were calculated (Table 2). In the three runs of butyric acid fermentation, the amounts of pretreated rice straw fed in the reactor were 1839, 1814, and 6779 g and the actual yields of butyric acid were 294, 317, and 1189 g, respectively, corresponding to 37 to 41% of the maximum theoretical yield based on the amount of rice straw fed into the reactor. Because the mixed culture produced carboxylic acids with different carbon chain lengths, the acetic acid equivalent (Aceq) was introduced to represent the amount of acetic acid that could have been produced if all the produced carboxylic acids were acetic acid (Fu and Holtzapple, 2011). The acetic acid equivalent α can be calculated from Equation (7).

(7) $$\begin{array}{lll} \text{Aceq}\alpha \;\left(\text{mol}/\text{L}\right) & = & \text{Acetic acid }\left(\text{mol}/\text{L}\right)\\ +\text{ Propionic acid }\left(\text{mol}/\text{L}\right)\times 1.75\\ +\text{ Butyric acid }\left(\text{mol}/\text{L}\right)\;\times \;2.5\\ +\text{ Valeric acid }\left(\text{mol}/\text{L}\right)\times 3.25\\ +\text{ Caproic acid }\left(\text{mol}/\text{L}\right)\times 4.0 \end{array}$$

**Table 2:** **The ratios of actual yield to theoretical yield of butyric acid fermentation in a stirred-tank reactor**.

	**Batch fermentation 1**	**Batch fermentation 2**	**Semi-continuous fermentation**
Cumulative rice straw fed (g)	1839	1814	6779
Theoretical yield of butyric acid (g)	790.8	780	2915
Actual yield of butyric acid (g)	293.6	316.9	1189.4
Actual yield of butyric acid/Theoretical yield (%)	37	41	41
Theoretical yield of total carboxylic acids (g)	1103.4	1088.4	4067.4
Actual yield of total carboxylic acids (g)	513.2	508.6	2047.7
Acetic acid equivalent (g)	764.7	774.3	3025.2
Actual yield of carboxylic acids/Theoretical yield (%)^a^	69	71	74

a The actual yield of carboxylic acids is represented by acetic acid equivalent.

On a mass basis, the acetic acid equivalent can be expressed as Equation (8).

(8) $$\begin{array}{l} \text{Aceq}\left(\text{g}/\text{L}\right)=\text{Aceq }\alpha \left(\text{mol}/\text{L}\right)\times 60 \end{array}$$

The measured carboxylic acids (including acetic, propionic, *i*-butyric, *n*-butyric, *i*-valyric, *n*-valyric, *i*-caproic and *n*-caproic acids) presented in Table 1 were converted to Aceq according to Equations (7, 8). Using this conversion, approximately 69 to 74% of the maximum theoretical yield of acetic acid was obtained. It is necessary to emphasize that the fermentation of monosaccharides by open mixed cultures may be expected to be completely converted to acetic acid with a carbon efficiency of 100% as presented in Equations (9, 10) (Holtzapple and Granda, 2009; Fu and Holtzapple, 2011).

(9) $$\begin{array}{l} {\text{C}}_{6}{\text{H}}_{12}{\text{O}}_{6}\to {3\text{ CH}}_{3}\text{COOH} \end{array}$$

(10) $$\begin{array}{l} {2\text{ C}}_{5}{\text{H}}_{10}{\text{O}}_{5}\to {5\text{ CH}}_{3}\text{COOH} \end{array}$$

If the above viewpoint holds, the ratios of actual yields to theoretical yields obtained in this study would be much lower than those of calculated using Equations (9, 10) instead of Equations (3, 5).

### Comparative assessment of butyric acid production by undefined mixed culture and pure culture

Despite its potential as a substitute for petroleum-derived butyric acid, the commercial production and application of biomass-derived butyric acid has been hindered by the high cost of microbial fermentation. To overcome this limitation, butyric acid fermentation has been extensively studied using various producers in combination with several substrates in batch, semi-continuous, continuous and cell-recycled modes (see Table 3). The presently preferred producers for butyric acid fermentation are the species of *Clostridium*, including *C. butyricum, C. tyrobutyricum, C. thermobutyricum, C. acetobutylicum*, and *C. populeti* (Zhang et al., 2009). *C. tyrobutyricum* is regarded as the most promising microorganism for industrial applications owing to its high productivity and selectivity (Dwidar et al., 2013). By improving the fermentation method, for example, immobilizing microbial cells to increase the biomass in the reactor and prevent the loss of cells greatly enhanced both the productivity and selectivity (Jiang et al., 2010); using the semi-continuous mode instead of the batch mode led to a higher final product concentration (Wei et al., 2013).

**Table 3:** **Summary of the results of butyric acid fermentation using undefined mixed culture and pure culture**.

**Butyric acid-producing strain**	**Substrate**	**Fermentor**	**Operation mode**	**Final titer (g/L)**	**Product selectivity (%)^a^**	**References**
*C. tyrobutyricum*	Glucose	5 L bioreactor	Suspended-cell	7.1	77.0	Jiang et al., 2010
			Immobilized-cell	13.7	81.6	
	Xylose		Suspended-cell	5.1	78.5	
			Immobilized-cell	10.1	82.9	
*C. tyrobutyricum*	Jerusalem artichoke hydrolysate	5 L fibrous-bed bioreactor	Repeated batch	27.5	N/A^b^	Huang et al., 2011
			Semi-continuous	60.4	98.8	
*C. tyrobutyricum*	Sugarcane bagasse hydrolysate	0.5 L fibrous-bed bioreactor	Batch	14.5	65.2	Wei et al., 2013
			Semi-continuous	20.9	76.8	
*C. tyrobutyricum*	Wheat straw hydrolysate	0.5 L vessel	Batch	9.9	76.7^c^	Liu et al., 2013
	Switchgrass hydrolysate			7.1	72.4^c^	
*C. tyrobutyricum* and *Bacillus* sp.	Sucrose	3 L bioreactor	Semi-continuous	34.2	92.8	Dwidar et al., 2013
*Thermobifida fusca*	Milled corn stover	5 L bioreactor	Batch	2.4	N/A^b^	Merklein et al., 2014
Undefined mixed culture	Sugarcane bagasse	1 L rotary centrifuge bottle	Continuous four-stage countercurrent	4.1	19.5	Thanakoses et al., 2003
Undefined mixed culture	NaOH pretreated rice straw	0.5 L serum bottle	Batch	6.0	75.9	Ai et al., 2014b
Undefined mixed culture	NaOH pretreated rice straw	21 L stirred-tank reactor	Batch	14.0–15.1	59.4–60.9	This study
			Semi-continuous	16.2	58.1	

a Percent of butyric acid from the total carboxylic acids.

b N/A, not available.

c The value was not available from the original reference, but was calculated from the data estimated from the figures.

Starchy substrate (such as corn) and simple sugars (such as molasses and glucose) are the traditional carbon sources for butyric acid fermentation. With the increasing price of the traditional feedstocks, attention turns to low-cost lignocellulosic biomass, which is considered a promising carbon source for industrial production of butyric acid. However, the pure culture of butyric acid-producing strains cannot utilize cellulose. *C. tyrobutyricum* can only utilize monosaccharides like glucose, xylose and fructose, even not disaccharides, such as sucrose and lactose. By co-culturing with a *Bacillus* sp. capable of producing a levansucrase enzyme, *C. tyrobutyricum* ATCC 25755 is able to ferment sucrose to butyric acid (Dwidar et al., 2013). To use lignocellulose as a carbon source, cellulolytic enzymes are required to break down substrate into single sugars that are further utilized by butyric acid-producing strain. It was reported that *C. thermobutyricum* was co-cultured with *C. thermocellum* for butyric acid production from cellulosic substrate (Dwidar et al., 2012). In the co-culture system, *C. thermocellum* produces cellulase and hemicellulase that hydrolyzes cellulose and hemicellulose into pentose and hexose sugars. Because of its higher growth rate and the quicker fermentation rate of pentose and hexose, *C. thermobutyricum* utilizes the monosaccharides liberated by *C. thermocellum* to produce butyric acid. A cellulolytic bacterium, *Thermobifida fusca*, was also reported to ferment a cellulosic substrate to butyric acid, in which 2.37 g/L of butyric acid was produced from milled corn stover (Merklein et al., 2014).

Using pure culture and defined mixed culture, the degradation efficiency of lignocellulosic feedstocks and butyric acid production are limited. By using undefined mixed culture, degradation efficiency is enhanced and supplementary cellulolytic enzymes are not necessary. A patented undefined mixed culture-based process, MixAlco™, has been developed to convert organic biomass to carboxylate acids (mainly acetic acid) which are then further converted to mixed alcoholic fuels (Granda et al., 2009; Holtzapple and Granda, 2009). The results from using the MixAlco™ process have demonstrated that undefined mixed culture is efficient at converting lignocellulosic biomass to carboxylic acids. A very few studies have been undertaken to investigate the production of butyric acid as the desired product using undefined mixed culture from lignocellulosic feedstocks, but suffering from low final product concentration and low selectivity.

A mixed culture produces mixed products. It has been revealed that the cellulose-degrading butyrate-producing mixed culture used in this study is composed of cellulolytic and xylanolytic bacteria, butyrate-producing bacteria and other acidogenic bacteria, including *Bacteroides cellulosilyticus, Clostridium cellulolyticum, Bacteroides graminisolvens, Bacteroides xylanisolvens, Cellulosilyticum ruminicola, Eubacterium xylanophilum, Clostridium polysaccharolyticum, Clostridium saccharolyticum, Thermincola carboxydiphila, Clostridium xylanolyticum, Clostridium algidixylanolyticum, Xylanibacter oryzae* (Ai et al., 2013). *n*-Butyric and acetic acids were the main liquid products, and another six carboxylic acids, including propionic, *i*-butyric, *i*-valeric, *n*-valeric, *i*-caproic and *n*-caproic, were detected in the fermentation broth. Therefore, high selectivity for the desired product is especially crucial for mixed culture-based processes.

The butyric acid production in the presence of an undefined mixed culture system is completed with the collaboration of cellulose- and hemicellulose-degrading bacteria, butyric acid-producing bacteria, acetic acid-producing bacteria and other bacteria. The cellulose- and hemicellulose-degrading bacteria provide substrate for acidogenic bacteria, and in return the acidogenic bacteria relieve substrate inhibition for cellulose- and hemicellulose-degrading bacteria. However, the bacteria groups with different functions require different growth and production conditions. As environmental factors change, the balance based on mutually beneficial relationships will likely be broken. The bacterial community structure shifts and hence the product spectrum varies. It was found that fermentation mode (batch or repeated-batch operation) greatly influences butyric acid production. The shift of microbial community structure is believed to be the reason behind the selectivity changes. When butyric acid production underwent a decrease, it was observed an increase in population of acetate- and valerate-producing bacteria and a decrease in butyrate-producing bacteria (Ai et al., 2014b). Thus, stability of microbial community structure is required for butyric acid production with undefined mixed culture.

Besides the growth and decline of population of byproduct-producing bacteria, the shift in metabolic pathway of butyrate-producing bacteria causes changes in selectivity of butyric acid production. In acidogenic fermentation systems, the microbes tend to produce acetic acid rather than butyric acid to meet their energy demands because more ATP is synthesized from the acetate metabolic pathway (Equation 3) than the butyrate metabolic pathway (Equation 4). In order for microorganisms to respond to adverse situations like pH decreases and excess NADH and H^+^, excreted acetic acid will be taken up and converted into butyric acid. Metabolism will then shift to butyric acid formation, which explains why the optimal pH for butyric acid fermentation is in the acidic pH range and why acetic acid formation peaked and then declined in the first 3 days (Ai et al., 2014a). As discussed above, low-pH stress induces microbes to produce butyric acid not acetic acid for less acidic end groups (-COOH). But neutral pH reduces the harm to microbial cell caused by undissociated carboxylic acids, and hence improves the substrate utilization and total carboxylic acids production. So, butyric acid production is optimal at neutral to slightly acidic pH range that maintains constant stress for butyric acid formation, but does not inhibit the microbial metabolism.

## Conclusions

A stirred-tank reactor was developed for butyric acid production from NaOH pretreated rice straw with a cellulolytic butyrate-producing microbial community. Of the two fermentation operations tested in this study, semi-continuous fermentation achieved the higher butyric acid productivity of 2.69 g/(L·d) with a concentration of 16.2 g/L. The actual yield of butyric acid corresponded to 41% of the maximum theoretical yield based on the amount of rice straw fed into the reactor. This study successfully demonstrated a CBP process for the bioconversion of low grade lignocellulosic biomass into butyric acid without supplementary cellulolytic enzymes.

## Author contributions

BA designed the experiment, operated the reactor and drafted the manuscript. XC and JM did the measurements and participated in data analysis. ZS, LZ, and XZ contributed in writing the draft. JL led and coordinated the overall project. All authors read and approved the final manuscript.

## Funding

This work was financially supported by the National Natural Science Foundation of China (Grant No. 51478141), Natural Science Foundation of Hainan Province of China (Grant No. 20154194) and State Key Laboratory of Urban Water Resource and Environment, Harbin Institute of Technology (Grant No. 2013DX11).

### Conflict of interest statement

The authors declare that the research was conducted in the absence of any commercial or financial relationships that could be construed as a potential conflict of interest.

## References

[B1] Agler M. T. Wrenn B. A. Zinder S. H. Angenent L. T. (2011). Waste to bioproduct conversion with undefined mixed cultures: the carboxylate platform. Trends Biotechnol. 29, 70–78. 10.1016/j.tibtech.2010.11.006 21190748

[B2] Ai B. Li J. Chi X. Meng J. Jha A. Liu C. (2014a). Effect of pH and buffer on butyric acid production and microbial community characteristics in bioconversion of rice straw with undefined mixed culture. Biotechnol. Bioprocess Eng. 19, 676–686. 10.1007/s12257-013-0655-z

[B3] Ai B. Li J. Chi X. Meng J. Liu C. Shi E. (2014b). Butyric acid fermentation of sodium hydroxide pretreated rice straw with undefined mixed culture. J. Microbiol. Biotechnol. 24, 629–638. 10.4014/jmb.1309.09078 24561721

[B4] Ai B. Li J. Song J. Chi X. Meng J. Zhang L. (2013). Butyric acid fermentation from rice straw with undefined mixed culture: enrichment and selection of cellulolytic butyrate-producing microbial community. Int. J. Agric. Biol. 15, 1075–1082.

[B5] Albuquerque M. G. E. Martino V. Pollet E. Avérous L. Reis M. A. M. (2011). Mixed culture polyhydroxyalkanoate (PHA) production from volatile fatty acid (VFA)-rich streams: effect of substrate composition and feeding regime on PHA productivity, composition and properties. J. Biotechnol. 151, 66–76. 10.1016/j.jbiotec.2010.10.070 21034785

[B6] Chan W. N. Fu Z. Holtzapple M. T. (2011). Co-digestion of swine manure and corn stover for bioenergy production in MixAlco™ consolidated bioprocessing. Biomass Bioenergy 35, 4134–4144. 10.1016/j.biombioe.2011.06.053

[B7] Cheng C.-L. Lo Y.-C. Lee K.-S. Lee D.-J. Lin C.-Y. Chang J.-S. (2011). Biohydrogen production from lignocellulosic feedstock. Bioresour. Technol. 102, 8514–8523. 10.1016/j.biortech.2011.04.059 21570833

[B8] Dwidar M. Kim S. Jeon B. S. Um Y. Mitchell R. J. Sang B.-I. (2013). Co-culturing a novel Bacillus strain with *Clostridium tyrobutyricum* ATCC 25755 to produce butyric acid from sucrose. Biotechnol. Biofuels 6:35. 10.1186/1754-6834-6-35 23452443 PMC3610116

[B9] Dwidar M. Park J.-Y. Mitchell R. J. Sang B.-I. (2012). The future of butyric acid in industry. Sci. World J. 2012, 1–9. 10.1100/2012/471417 22593687 PMC3349206

[B10] Forrest A. K. Sierra R. Holtzapple M. T. (2010). Suitability of pineapple, Aloe vera, molasses, glycerol, and office paper as substrates in the MixAlco process™. Biomass Bioenergy 34, 1195–1200. 10.1016/j.biombioe.2010.03.013

[B11] Fu Z. Holtzapple M. T. (2010). Consolidated bioprocessing of sugarcane bagasse and chicken manure to ammonium carboxylates by a mixed culture of marine microorganisms. Bioresour. Technol. 101, 2825–2836. 10.1016/j.biortech.2009.11.104 20044250

[B12] Fu Z. Holtzapple M. T. (2011). Anaerobic thermophilic fermentation for carboxylic acid production from in-storage air-lime-treated sugarcane bagasse. Appl. Microbiol. Biotechnol. 90, 1669–1679. 10.1007/s00253-011-3178-6 21365471

[B13] Granda C. B. Holtzapple M. T. Luce G. Searcy K. Mamrosh D. L. (2009). Carboxylate platform: the MixAlco process Part 2: process economics. Appl. Biochem. Biotechnol. 156, 107–124. 10.1007/s12010-008-8481-z 19184549

[B14] Hao X. Zhou M. Yu H. Shen Q. Lei L. (2006). Effect of sodium ion concentration on hydrogen production from sucrose by anaerobic hydrogen-producing granular sludge. Chin. J. Chem. Eng. 14, 511–517. 10.1016/S1004-9541(06)60106-7

[B15] Holtzapple M. T. Granda C. B. (2009). Carboxylate platform: the MixAlco process Part 1: comparison of three biomass conversion platforms. Appl. Biochem. Biotechnol. 156, 95–106. 10.1007/s12010-008-8466-y 19127445

[B16] Huang J. Cai J. Wang J. Zhu X. Huang L. Yang S.-T.. (2011). Efficient production of butyric acid from Jerusalem artichoke by immobilized *Clostridium tyrobutyricum* in a fibrous-bed bioreactor. Bioresour. Technol. 102, 3923–3926. 10.1016/j.biortech.2010.11.112 21169015

[B17] Jiang L. Wang J. Liang S. Wang X. Cen P. Xu Z. (2010). Production of butyric acid from glucose and xylose with immobilized cells of *Clostridium tyrobutyricum* in a fibrous-bed bioreactor. Appl. Biochem. Biotechnol. 160, 350–359. 10.1007/s12010-008-8305-1 18651247

[B18] Lee J. M. Upare P. P. Chang J. S. Hwang Y. K. Lee J. H. Hwang D. W.. (2014). Direct hydrogenation of biomass-derived butyric acid to n-butanol over a ruthenium-tin bimetallic catalyst. ChemSusChem 7, 2998–3001. 10.1002/cssc.201402311 25123894

[B19] Liu S. Bischoff K. M. Leathers T. D. Qureshi N. Rich J. O. Hughes S. R. (2013). Butyric acid from anaerobic fermentation of lignocellulosic biomass hydrolysates by *Clostridium tyrobutyricum* strain RPT-4213. Bioresour. Technol. 143, 322–329. 10.1016/j.biortech.2013.06.015 23811065

[B20] Merklein K. Fong S. S. Deng Y. (2014). Production of butyric acid by a cellulolytic actinobacterium *Thermobifida fusca* on cellulose. Biochem. Eng. J. 90, 239–244. 10.1016/j.bej.2014.06.012

[B21] Olson D. G. McBride J. E. Shaw A. J. Lynd L. R. (2012). Recent progress in consolidated bioprocessing. Curr. Opin. Biotechnol. 23, 396–405. 10.1016/j.copbio.2011.11.026 22176748

[B22] Richter H. Qureshi N. Heger S. Dien B. Cotta M. A. Angenent L. T. (2012). Prolonged conversion of n-butyrate to n-butanol with *Clostridium saccharoperbutylacetonicum* in a two-stage continuous culture with in-situ product removal. Biotechnol. Bioeng. 109, 913–921. 10.1002/bit.24380 22095002

[B23] Temudo M. F. Mato T. Kleerebezem R. van Loosdrecht M. C. M. (2009). Xylose anaerobic conversion by open-mixed cultures. Appl. Microbiol. Biotechnol. 82, 231–239. 10.1007/s00253-008-1749-y 19015850 PMC7419444

[B24] Thanakoses P. Mostafa N. Holtzapple M. (2003). Conversion of sugarcane bagasse to carboxylic acids using a mixed culture of mesophilic microorganisms. Appl. Biochem. Biotechnol. 107, 523–546. 10.1385/ABAB:107:1-3:523 12721433

[B25] Van Soest P. J. Robertson J. B. Lewis B. A. (1991). Methods for dietary fiber, neutral detergent fiber, and nonstarch polysaccharides in relation to animal nutrition. J. Dairy Sci. 74, 3583–3597. 10.3168/jds.S0022-0302(91)78551-2 1660498

[B26] Wei D. Liu X. Yang S.-T. (2013). Butyric acid production from sugarcane bagasse hydrolysate by *Clostridium tyrobutyricum* immobilized in a fibrous-bed bioreactor. Bioresour. Technol. 129, 553–560. 10.1016/j.biortech.2012.11.065 23270719

[B27] Zhang C. Yang H. Yang F. Ma Y. (2009). Current progress on butyric acid production by fermentation. Curr. Microbiol. 59, 656–663. 10.1007/s00284-009-9491-y 19727942

